# Transcriptome sequencing and metabolome analysis reveal the molecular mechanism of *Salvia miltiorrhiza* in response to drought stress

**DOI:** 10.1186/s12870-024-05006-7

**Published:** 2024-05-23

**Authors:** Ying Zhou, Yan-Hong Bai, Feng-Xia Han, Xue Chen, Fu-Sheng Wu, Qian Liu, Wen-Zhe Ma, Yong-Qing Zhang

**Affiliations:** 1 State Key Laboratory of Quality Research in Chinese Medicine, Macau University of Science and Technology, Macau, China; 2 College of Pharmacy, Shandong University of Traditional Chinese Medicine, Jinan, China; 3 Key Laboratory of Traditional Chinese Medicine Classical Theory, Ministry of Education, Jinan, China; 4 Shandong Provincial Center of Forest and Grass, Jinan, China

**Keywords:** *Salvia miltiorrhiza*, Drought stress, Transcriptome, Metabolome

## Abstract

*Salvia miltiorrhiza* is commonly used as a Chinese herbal medicine to treat different cardiovascular and cerebrovascular illnesses due to its active ingredients. Environmental conditions, especially drought stress, can affect the yield and quality of *S. miltiorrhiza.* However, moderate drought stress could improve the quality of *S. miltiorrhiza* without significantly reducing the yield, and the mechanism of this initial drought resistance is still unclear. In our study, transcriptome and metabolome analyses of *S. miltiorrhiza* under different drought treatment groups (CK, A, B, and C groups) were conducted to reveal the basis for its drought tolerance. We discovered that the leaves of *S. miltiorrhiza* under different drought treatment groups had no obvious shrinkage, and the malondialdehyde (MDA) contents as well as superoxide dismutase (SOD) and peroxidase (POD) activities dramatically increased, indicating that our drought treatment methods were moderate, and the leaves of *S. miltiorrhiza* began to initiate drought resistance. The morphology of root tissue had no significant change under different drought treatment groups, and the contents of four tanshinones significantly enhanced. In all, 5213, 6611, and 5241 differentially expressed genes (DEGs) were shared in the A, B, and C groups compared with the CK group, respectively. The results of KEGG and co-expression analysis showed that the DEGs involved in plant-pathogen interactions, the MAPK signaling pathway, phenylpropanoid biosynthesis, flavonoid biosynthesis, and plant hormone signal transduction responded to drought stress and were strongly correlated with tanshinone biosynthesis. Furthermore, the results of metabolism analysis indicated that 67, 72, and 92 differentially accumulated metabolites (DAMs), including fumarate, ferulic acid, xanthohumol, and phytocassanes, which were primarily involved in phenylpropanoid biosynthesis, flavonoid biosynthesis, and diterpenoid biosynthesis pathways, were detected in these groups. These discoveries provide valuable information on the molecular mechanisms by which *S. miltiorrhiza* responds to drought stress and will facilitate the development of drought-resistant and high-quality *S. miltiorrhiza* production.

**Supplementary Information:**

The online version contains supplementary material available at 10.1186/s12870-024-05006-7.

## Introduction

Drought stress, one of the worst environmental stressors, has serious impacts on the distribution of species, the ecological environment and the development of productivity [1, 2]. Drought is a common abiotic stress and affects plant growth and development. Extreme drought stress has a major impact on a variety of physiological and biochemical processes of plants, leading to cell dehydration and internal environment disorders, inhibition of plant photosynthesis and enzyme activity, and reactive oxygen species (ROS) accumulation [3, 4]. Moreover, due to their high reactivity, excess ROS are hazardous and can destroy nucleic acids, proteins, and lipids [5]. In addition, severe drought stress could reduce yield and quality, which in turn affect real productivity [6].

Adversity produces quality. Under moderate drought stress, the yield may be reduced, but the quality will be improved, especially for medicinal plants [7]. That's because, in the early stage of drought stress, plants have evolved a series of response mechanisms, including cellular modifications and physiological and metabolic changes [8, 9]. To meet the challenges posed by drought stress, plants can stabilize cell structure and protein activity and accumulate osmotic-regulating substances, such as soluble sugar and proline, thus improving their ability to produce ROS [10]. Furthermore, the accumulation of flavonoids, one type of widely distributed secondary metabolites, can relieve the damage of ROS to plants [11, 12]. In addition, phytohormones, such as abscisic acid (ABA) and jasmonate (JA), are crucial in the response to drought stress [13]. It is well known that through the regulation of stress-responsive genes, ABA can stimulate short-term responses, including stomatal closure, and lead to the maintenance of water balance and longer-term growth responses [14]. Numerous regulators in the JA signaling pathway are connected to drought stress responses according to previous studies. Regulators usually do not have independent regulatory roles but often combine to form a complex signaling network [15]. The extent of drought is gradually expanding due to global climate change, and an increasing number of studies have focused on how plants adapt to drought stress. Therefore, it will be helpful to improve the value of medicinal plants to reveal the possible mechanism of drought stress response by means of combined analysis of omics.

In the study of plant genetics, RNA-seq has been widely employed for a variety of purposes since the introduction of high-throughput sequencing technology, particularly transcriptome analysis, which is used to identify differentially expressed genes (DEGs) in distinct biological processes [16]. Metabolite profiling has been extensively employed to investigate the alterations in metabolites caused by genetic modification and environmental factors [17]. It is common to use UPLC/ESI-Q TRAP-MS/MS to identify and evaluate plant metabolites, and this technology has been extensively used to examine metabolites in various species, such as tomato [18]. From a molecular perspective, plants participate in the response to drought stress by regulating their metabolic pathways and activating relevant signaling networks. In *Casuarina equisetifolia,* for example, a total of 5033 and 8159 DEGs were identified with transcriptome analysis after different periods of drought stress treatment, and they were primarily involved in flavonoid and phenylpropanoid biosynthesis as well as plant hormone signal transduction. Moreover, a metabolomic study revealed that the contents of amino acids, phenolic acids, and flavonoids were also increased [19]. In addition, 2451 DEGs and 354 differentially accumulated metabolites (DAMs) were found under drought treatment in *Pohlia nutans.* Combining transcriptome and metabolomic analyses, researchers have hypothesized that *P. nutans* strongly relies on the plant hormone signaling pathway and flavonoid metabolism pathway, as well as stress-related genes involved in these pathways, such as *NCED3, PP2C,* and *PYL,* which are involved in the ABA signaling pathway. In addition, stress-related genes also included *AOS* and *JAZ* in the JA signaling pathway, *CHS, FLS, FNS,* and *UFGT* in the flavonoid pathway, and transcription factors (*ERF* and *DRE*B) [20].

*Salvia miltiorrhiza* Bunge, also referred to as “Danshen”, is a widely studied medicinal plant. The medicinal parts of *S. miltiorrhiza*, especially the tanshinones, are mainly derived from its roots, which are frequently utilized to treat cardiovascular and cerebrovascular diseases [21, 22]. Increasingly harsh environmental conditions, especially global warming, gradually aggravate drought, which has caused serious harm to the yield and quality of *S. miltiorrhiza* [23, 24]. Studies have shown that moderate drought stress could improve the quality of medicinal plants without reducing the yield [7]. Therefore, it is of great significance to study the response mechanism under moderate drought stress and this will help to improve the drought stress resistance and adaptability of *S. miltiorrhiza*. Although the genome sequence, transcriptome, and metabolome from different development periods [25–27] and different tissues [28], tissue cultures from various inductions [29], and explanations for phenotypic changes [30] in *S. miltiorrhiza* have been obtained, little research has been executed on the transcriptome and metabolome responses to drought stress. Consequently, to explore the drought response of *S. miltiorrhiza*, a comprehensive analysis of transcriptomic and metabolomic data was performed in this study. We identified the genes and metabolites that were altered under drought stress. This study provides insight into the molecular basis of *S. miltiorrhiza* drought stress resistance. This could serve as a theoretical foundation for further research on the molecular mechanism and genetic regulation of drought stress resistance in *S. miltiorrhiza* under drought stress.

## Materials and methods

### Plant materials and experimental treatments

The seedlings of *Salvia miltiorrhiza* Bunge (‘Huadan No.2’) identified by the professor Q. L. were grown in a green house (temperature: 18~28℃; relative humidity: 60%~80%) at the Medicinal Herb Garden, Shandong University of Traditional Chinese Medicine. After three months, the seedlings with consistent growth and root lengths of approximately 12~15 cm were chosen and used for pot cultivation with three seedlings per pot. The potting soil, which was composed of surface soil, nutrient soil and fine river sand (the ratio was 3:1:2), was contained in 25 cm (height) × 22 cm (inner diameter) plastic buckets.

For drought treatment*, S. miltiorrhiza* seedlings with essentially the same growth trend were chosen in our study. Four treatment groups were set up in this experiment. The soil water content was 75% (75% θf), 65% (65% θf), 55% (55% θf), and 45% (45% θf) of the maximum field water capacity, corresponding to CK (the control group), A, B, and C, respectively [31, 32]. There were 150 seedlings in total and 30 seedlings in each group. The treated *S. miltiorrhiza* seedlings were irrigated regularly to ensure that the soil moisture content remained at the set gradient in these four groups. The soil was weighed with an electronic scale at 17:00 every day, and the missing water was made up. The treatment was continued for 30 days. The roots of *S. miltiorrhiza* were frozen using liquid nitrogen and stored at -80 ℃ for extraction of total RNA, tanshinone content measurement, mass spectrometry imaging analysis, and multi-omics analysis. The leaves of *S. miltiorrhiza* were frozen using liquid nitrogen for measurement of physiological indexes. In our research, three biological replicates of each experiment were performed.

### Measurements of physiological indexes and tanshinone content

To analyse the changes in physiological indexes of *S. miltiorrhiza* plants under drought stress, total protein, superoxide dismutase (SOD), peroxidase (POD), malondialdehyde (MDA), proline (PRO), and catalase (CAT) were determined using commercial kits purchased from the Jiancheng Bioengineering Institute (Nanjing, China) [33–35]. Table S 1 in the supplementary material displays all of the measurement method of these physiological indexes.

The main tanshinone contents in the roots of *S. miltiorrhiza* plants treated with different drought stress were determined using high performance liquid chromatography (HPLC, Waters 2695, USA). It was performed according to the previous method in our laboratory. All of the roots of samples were freeze-dried for 48 h, and then grind to a powder using a mortar. The 0.25g sample was extracted in 25 mL 100% methyl alcohol and subjected to ultrasonic shock for 50 min. The extracts were centrifuged at 4000 rpm for 10 min and later filtered through a 0.22 μm microporous membrane (Jinteng, Tianjin China) for analysis.

### Mass spectrometry imaging analysis

The roots of *S.miltiorrhiza* plants were removed from the ultra-low temperature refrigerator at -80 °C and placed in an incubator at -20 °C for rewarming for 2 h. Leica Cryo-Gel was used to fix the tissue on the sample holder of the microtome. Then 15 μm thick frozen sections were prepared at -20 °C using a Thermo CryoStar NX50 NOVPD cryotome. First, the sections were placed in a -20 °C desiccator and vacuumized for 1 h, then kept at room temperature and vacuumized for 6 h. Finally, the sections were fixed in plant tissue fixation solution for 10 min and rinsed with water for 1 min. The sections were put into solid green dye solution for 5-10 min, and then washed with water to remove excess dye solution. Sections were successively immersed in 50%, 70% and 80% gradient alcohol for 3-5s; The sections were immersed in solid green dye for 30-60 s and dehydrated in three cylinders of absolute ethanol. The slices were immersed in clean xylene and transparent for 5 min. The slices were sealed with neutral gum. The images were examined under a microscope and subsequently analyzed.

### Transcriptome sequencing and data analysis

First, total RNA of our samples was extracted using the FastPure Universal Plant Total RNA Isolation Kit (Vazyme, Nanjing, China). Then, cDNA was synthesized using the PrimeScript^TM^ RT Reagent Kit (TaKaRa, Japan). The integrity of the RNA was detected through agarose gel electrophoresis (AGE), and the concentration of RNA was measured using a nucleic acid spectrometer (Thermo Scientific, USA). Poly (A) mRNA was enriched from the total RNA using oligo (dT) magnetic beads. The sequencing adaptors were attached to segments of acceptable length that had undergone end repair, and a poly(A) tail was added. Sequencing was performed using a BGISEQ-500 high-throughput gene sequencing platform (MGI, China).

### Quantitative real-time PCR (qRT‒PCR) analysis

The technique outlined above was used to extract total RNA. Following the manufacturer’s instructions, total RNA was subsequently reverse transcribed into cDNA using an RT Reagent Kit. Quantitative real-time PCR (qRT-PCR) was carried out with TB Green Premix Ex Taq^TM^ II (TaKaRa, Japan) using a CFX96 Real-Time PCR System (Bio-Rad, United States). In this study, all qRT-PCR data were normalized to β-actin. For each biological replicate, each experiment was run in triplicate. The gene relative expression levels were calculated using the 2^-∆∆Ct^ method [36]. Table S 2 in the supplementary material displays all of the primer sequences.

### Ultra-performance liquid chromatography (UPLC) parameters and ESI-Q trap-MS/MS

An LC-ESI-MS/MS system (HPLC, Shim-pack UFLC SHIMADZU CBM30A system; MS, Applied Biosystems 6500 Q TRAP) was employed to evaluate the sample extracts. The following analytical conditions were applied: (1) HPLC: the column was a Waters ACQUITY UPLC HSS T3 C18 (1.8 μm, 2.1 mm*100 mm); the solvent system included water and acetonitrile with 0.04% acetic acid added to each. On a triple Q TRAP, API 6500 Q TRAP LC/MS/MS system with an ESI Turbo Ion-Spray interface, working in a positive ion mode and managed by Analyst 1.6.3 software (AB Sciex), linear ion hydrazine-flight time (LIT) and triple quadrupole (QQQ) scans were obtained [37].

### Coexpression analysis

After integrating data from these two major omics approaches, coexpression network analysis was conducted with R > 0.8 as the input file. Finally, the coexpression network was visualized through Cytoscape v3.8.0.

### Statistical assessment

All of the experimental results in our research were reviewed using a t-test with a p value below 0.05. Additionally, SPSS 22.0 was used to execute all statistical analyses.

## Results

### The effects of drought stress on the above-ground parts of *S. miltiorrhiza*

**Fig. 1:**
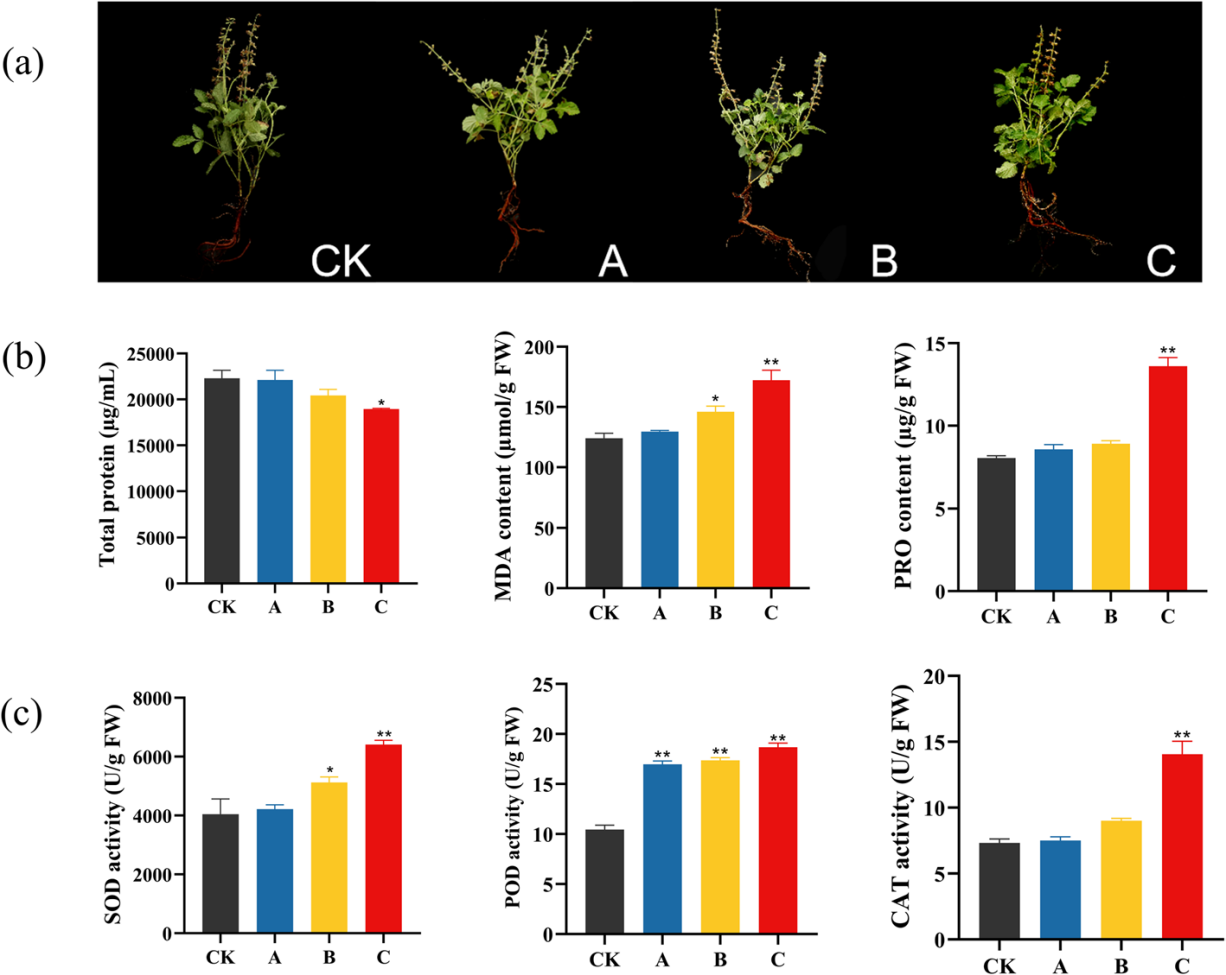
Effects of drought stress on the aboveground *S. miltiorrhiza*. **a** Phenotypic changes in *S. miltiorrhiza* after different drought stress treatments. **b** The contents of total protein, MDA, and PRO under drought stress. **c** The activity of different physiological indexes under drought stress. Values are presented as the means±SDs with three replicates. The symbols “**” and “*” represent *p* values below 0.01 and 0.05, respectively

When severe drought stress occurs, the above-ground parts of the plant are the first to show changes, such as leaf wilting [38]. Therefore, we first observed the changes in the above-ground parts of *S. miltiorrhiza* and we found the leaves of *S. miltiorrhiza* after drought stress treatment had no obvious shrinkage, the stems were straight in comparison to those in the CK group (Fig. 1a). The changes of physiological indexes were measured to determine the impact of drought stress on the physiology of *S. miltiorrhiza*. With increasing levels of drought stress, the contents of MDA and PRO gradually increased, reaching their highest levels in group C (increased by 1.37-fold and 1.66-fold, respectively). However, the content of total protein decreased gradually (Fig. 1b). Moreover, the activities of SOD, POD, and CAT, which increased by 1.43, 1.87, and 1.88 times, respectively, also improved with increasing drought degree in group C (Fig. 1c). These results indicate that the method of our drought stress treatment is moderate. It has no adverse effects on the growth and development of *S. miltiorrhiza* under moderate drought stress, and the drought-resistant response has been initiated in the leaves of *S. miltiorrhiza*.

### The effects of drought stress on the roots of *S. miltiorrhiza*

**Fig. 2:**
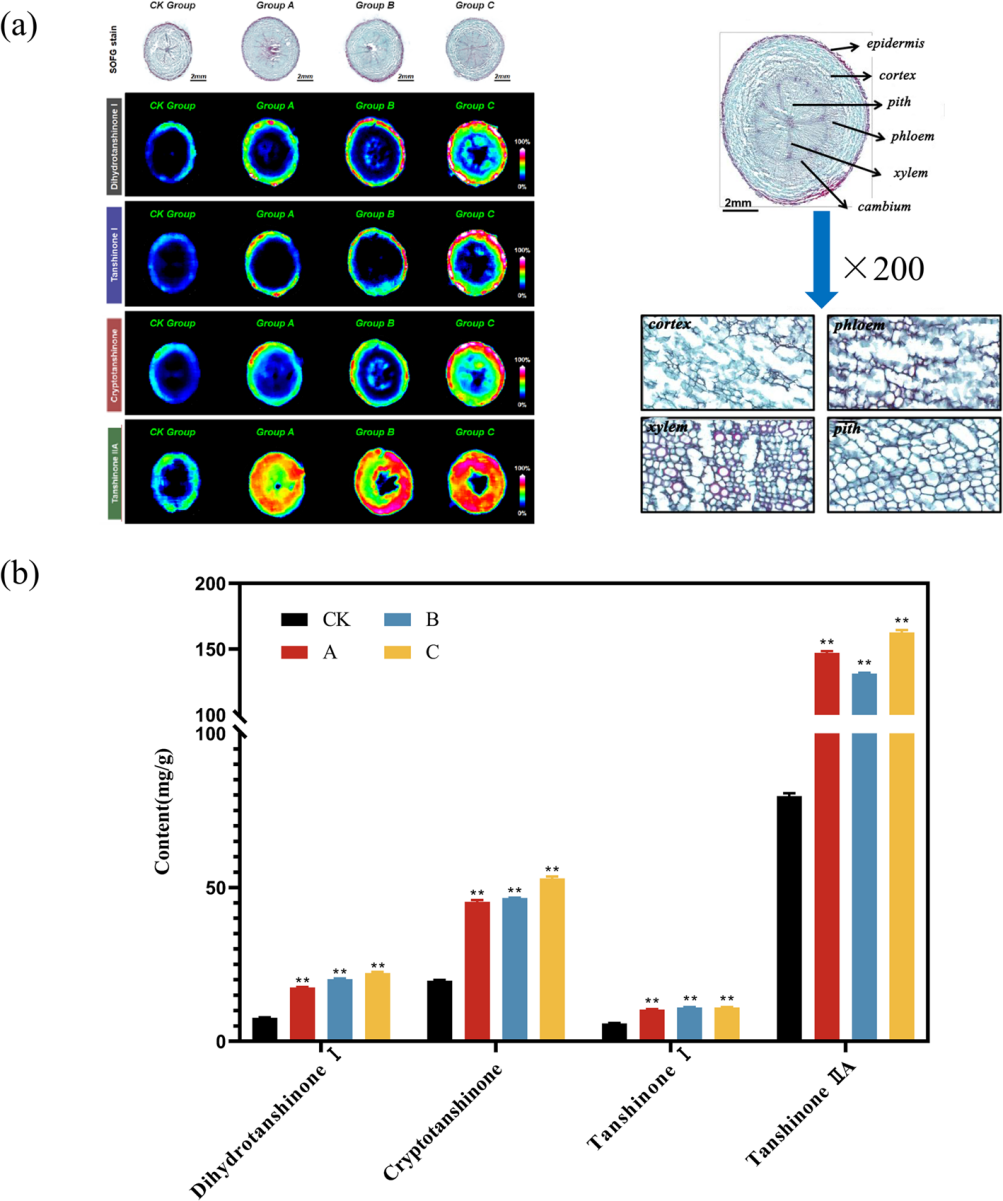
Effects of drought stress on the roots of *S. miltiorrhiza*. **a** The mass spectroscopic image of four tanshinones in root tissue and tissue sections in the root under drought stress. The closer the color is to red, the higher the tanshinone content. **b** Content changes of four tanshinones in roots of *S. miltiorrhiza* plants under drought stress*.* The symbols “**” and “*” represent *p* values below 0.01 and 0.05, respectively

As the root of *S. miltiorrhiza* is the main source of medicinal ingredients, meanwhile, we also observed the morphological changes of the roots using the mass spectrometry imaging technology. We found that the tissue structure, including epidermis, cortex, pith, phloem, xylem, and cambium, were not severely affected in comparison to those in the CK group (Fig. 2a). In addition, we also found that the component distribution, including dihydrotanshinone I, cryptotanshinone, tanshinone I, and tanshinone IIA were mostly distributed in the peripheral part of roots, while low in the central pith and the contents of them were higher with increasing levels of drought stress. To further verify the effect of drought stress on the accumulation of tanshinones, the contents of four tanshinones in the roots were determined using HPLC technology. All of them showed a trend of gradual increase with the increase of drought degree. In the highest group C, the content of the four active ingredients was 21.95 mg/g, 52.47 mg/g, 13.96 mg/g and 161.26 mg/g, which increased 2.89-, 2.68-, 2.39- and 2.04-fold compared with CK, respectively (Fig. 2b). These results indicate that moderate drought stress would not adversely affect the tissue structure and component distribution of the roots in *S. miltiorrhiza*. Simultaneously, moderate drought stress could promote the accumulation of tanshinone components.

### Differentially expressed genes (DEGs) under different degrees of drought stress

Transcriptomes were studied to identify DEGs in the samples to understand the molecular response of *S. miltiorrhiza* to drought stress. The CK, A, B, and C groups produced a total of 164.77 M, 159.52 M, 157.35 M, and 161.27 M raw reads, respectively. Both Q20 and Q30 had values greater than 88%, implying that the data quality could be utilized for further investigation (Table S 3). In addition, heatmap analysis showed that all of these groups’ correlation values were higher than 0.9 (Figure S 1). The principal component analysis (PCA) results showed that the differences in Unigene expression under distinct drought stress treatments were significant (Figure S 2). Therefore, we subsequently examined the gene expression in these groups under various degrees of drought stress.

**Fig. 3:**
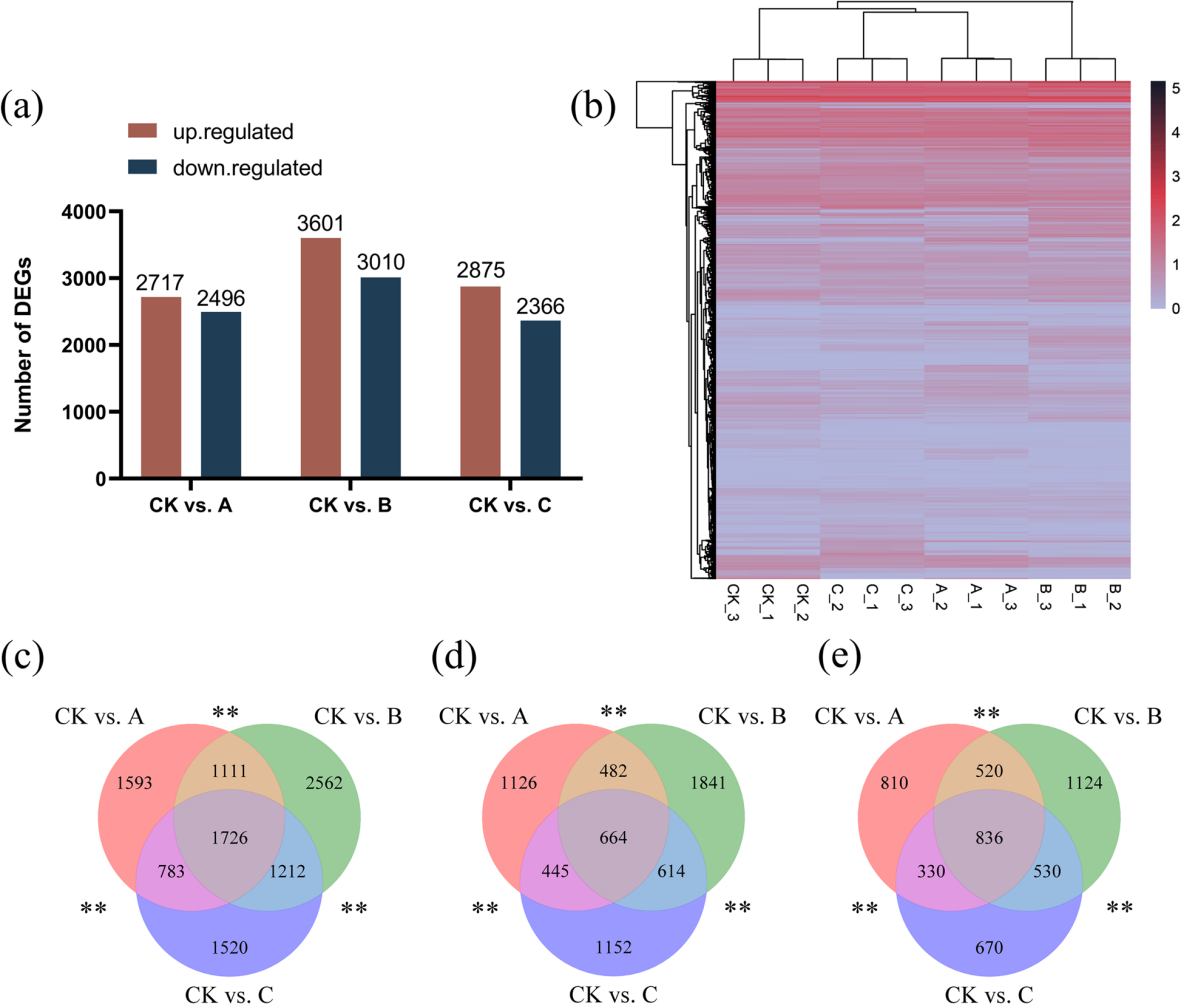
Identification of DEGs and transcriptome analysis. (**a**) The number of genes that were up- and down-regulated under various degrees of drought stress. (**b**) Heatmaps of DEGs compared between different groups. (**c**, **d**, **e**) Venn diagram of DEGs. (**c**) All differentially expressed genes (DEGs), (**d**) DEGs that were upregulated, and (**e**) DEGs that were downregulated. Values represent the difference in DEGs between pairs. The symbols “**” and “*” represent *p* values below 0.01 and 0.05, respectively

The significance of variations in gene expression was assessed using rigorous thresholds of FDR < 0.01 and log_2_FC ≥ 1. A total of 5213, 6611, and 5241 genes were differentially expressed in CK vs. A, CK vs. B, and CK vs. C, respectively, and there were more up-regulated genes than down-regulated genes (Fig. 3a). The top 15 genes with up-regulated and down-regulated differential expression are listed in Table S 4. The variations in DEGs between these groups are represented by Venn diagrams. In the aggregate, 1726 genes were identified, with 664 genes commonly upregulated and 836 genes commonly downregulated. Moreover, it was discovered that there were 1111 common genes identified in the CK vs. A and CK vs. B groups, 783 common genes identified between the CK vs. A and CK vs. C groups, and 1212 common genes identified in the CK vs. B and CK vs. C groups. (Fig. 3c, d, e). The heatmap in Fig. 3b displays the expression of DEGs in the comparative groups.

### Gene Ontology (GO) enrichment analysis of *S. miltiorrhiza* in response to different degrees of drought stress

**Fig. 4:**
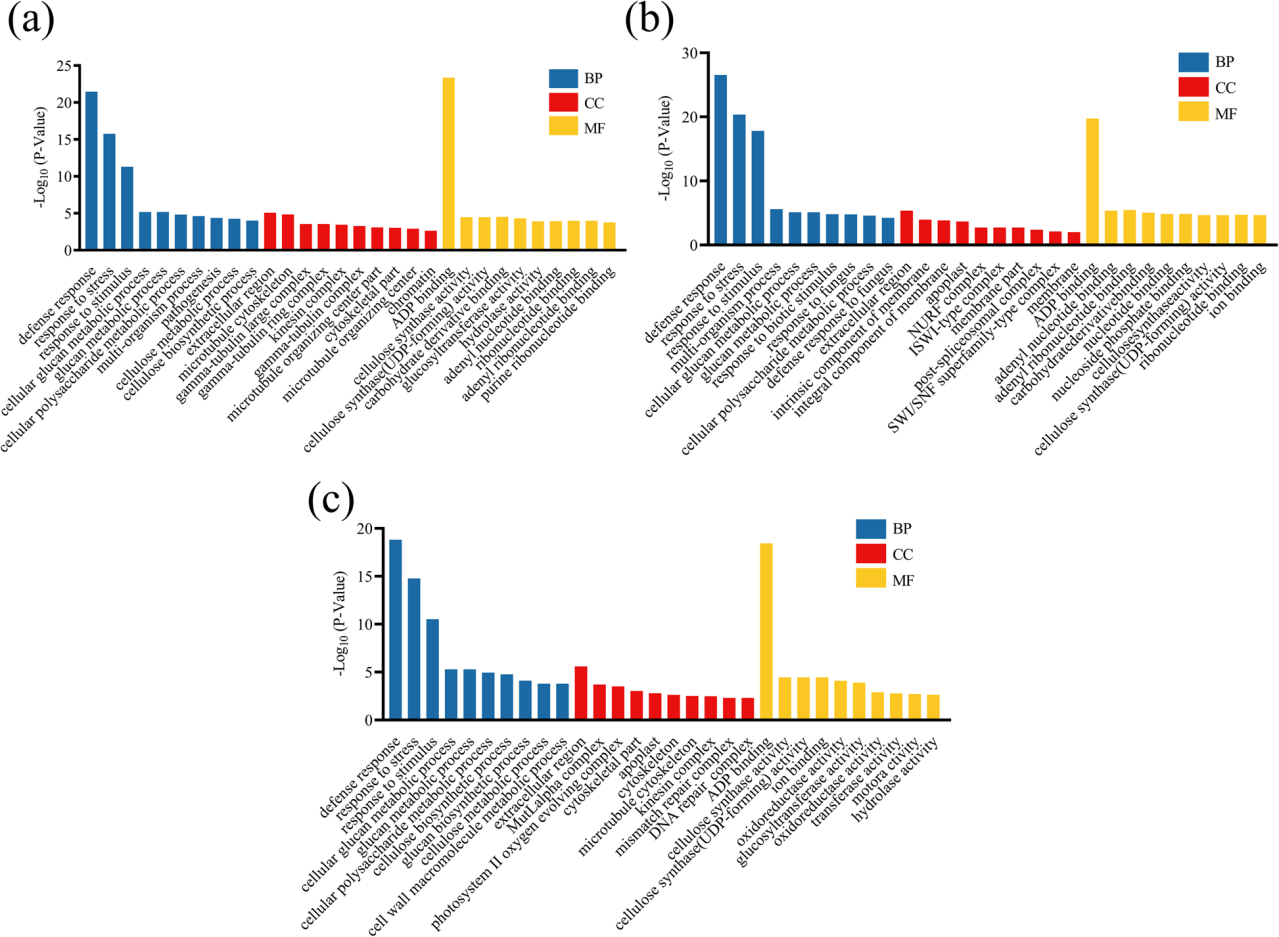
Distribution and GO enrichment analysis of DEGs under different degrees of drought stress. (**a**) CK vs. A, (**b**) CK vs. B, (**c**) CK vs. C

Over the years, Gene Ontology (GO) enrichment analysis has been broadly performed to annotate gene function and determine gene enrichment [39]. It is usually used to describe the functions of DEGs obtained from RNA-seq at three main levels: molecular function (MF), cellular component (CC) and biological process (BP). In our study, GO analysis indicated that in groups A, B, and C, more than half of the DEGs were categorized as “defense response”, “response to stress” and “response to stimulus” belonging to the BP category, “extracellular region” in the CC category, and “ADP binding” and “cellulose synthase activity” in the MF category. In addition, many DEGs were annotated under various metabolic processes in the BP category and various synthase activities in the MF category (Fig. 4a, b, c). According to these results, *S. miltiorrhiza* may enhance drought resistance by increasing a variety of synthase activities and various metabolic processes. TopGO analysis further revealed that the BP (GO: 0008150), CC (GO: 0005575), and MF (GO: 0003674) in these groups were the most significantly enriched terms in these three categories (Figure S 3).

### Kyoto Encyclopedia of Genes and Genomes (KEGG) pathway enrichment analysis of *S. miltiorrhiza* in response to different degrees of drought stress

**Fig. 5:**
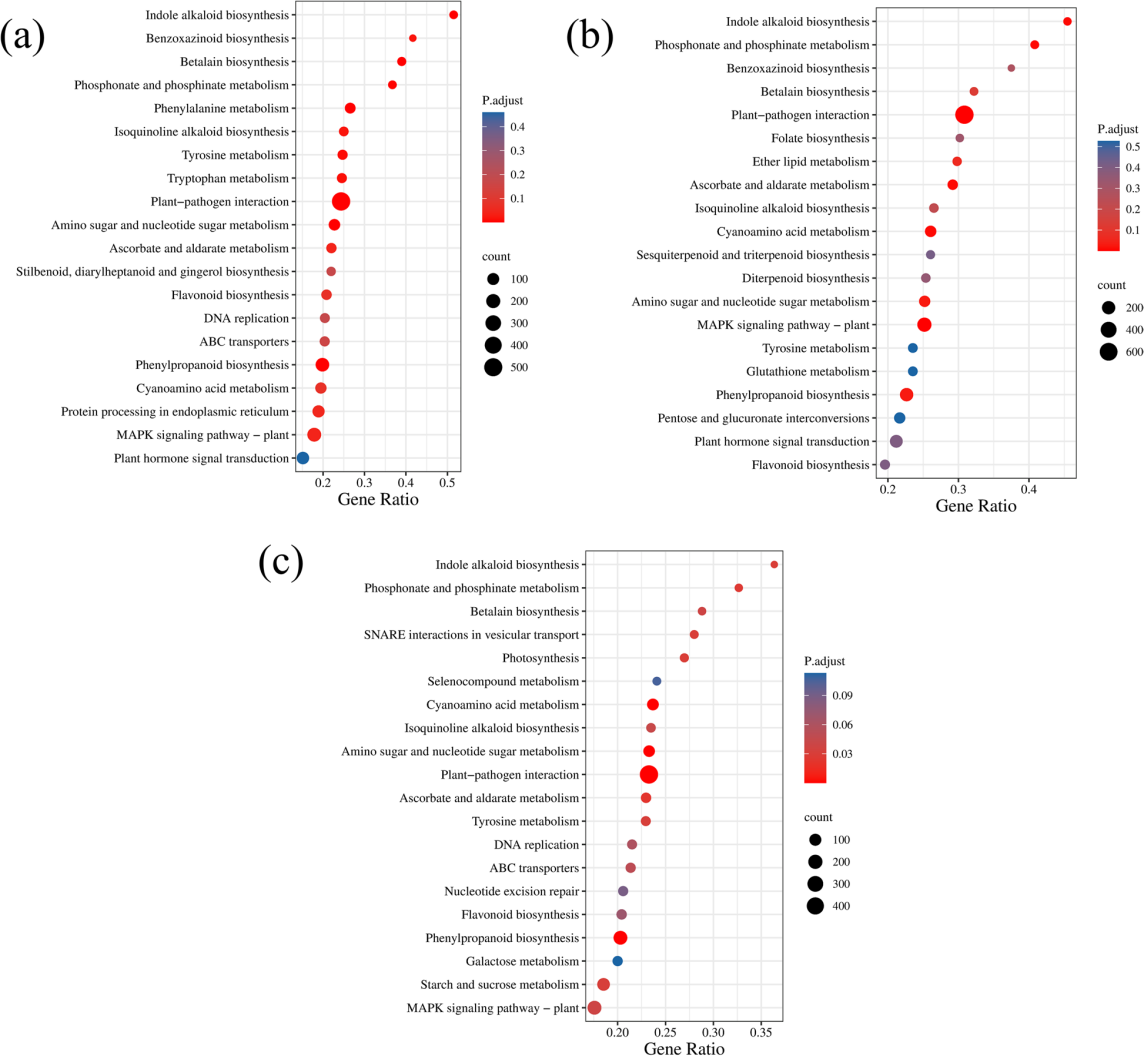
KEGG analysis of DEGs after different degrees of drought stress. **a**-**c** represent three different treatment groups compared with control groups. The gene ratio is the proportion of DEGs to all of the genes that have been noted in this pathway. The images are used and adapted with the permission of Kanehisa Laboratories

To better comprehend the biological functions and gene interactions, 2185 of 14606 DEGs from CK vs. A, 2747 of 14606 from CK vs. B, and 2139 of 14606 from CK vs. C were selected, and the most abundant metabolic pathways were examined. Our results indicated that all DEGs involved in plant-pathogen interaction (ko04626), phenylpropanoid biosynthesis (ko00940), and MAPK signaling pathway (ko04016) were significantly enriched by different degrees of drought stress (Fig. 5). In addition, we found that these DEGs were also prevalent in flavonoid biosynthesis (ko00941). Notably, among the A and B groups compared with the CK group, these genes were also strongly prevalent in plant hormone signal transduction (ko04075). Moreover, among CK vs. B, these DEGs were also highly abundant in diterpenoid biosynthesis (ko00904). These results showed a possible connection between *S. miltiorrhiza* drought resistance and the DEGs implicated in these pathways.

### Analysis of differentially expressed TFs

**Fig. 6:**
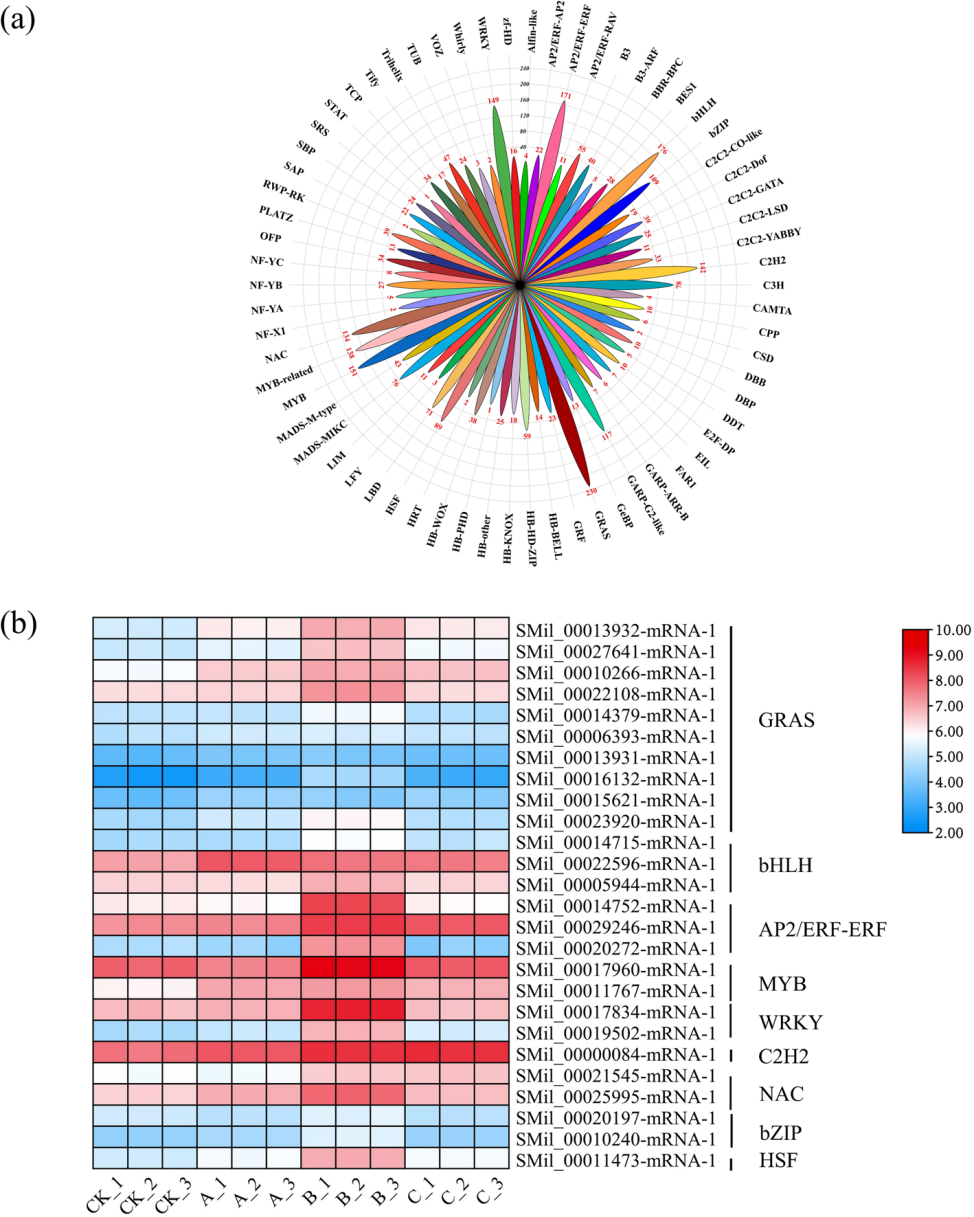
Changes in TFs under different degrees of drought stress. **a** Distribution of TFs. **b** DEGs assigned to TFs under drought stress treatment. GRAS: GRAS-domain transcription factors; bHLH: basic helix-loop-helix transcription factor; AP2/ERF-ERF: APETALA2/ethylene-responsive element binding factors-ethylene-responsive element binding factors; MYB: MYB-related transcription factors; WRKY: WRKY transcription factors; C2H2: C2H2 type zinc finger; NAC: NAC (NAM, ATAF1, 2, CUC2) transcription factors; bZIP: basic region/leucine zipper; HSF: heat stress transcription factor

In addition to aiding in plant growth and development, transcription factors (TFs) also influence how plants respond to abiotic stress. In our research, TFs identified in whole groups were evaluated to elucidate their biological functions under drought stress. Through the comparison of the databases, we revealed that there were 2760 DEGs encoding TFs. Among them, 66 families were classified, including GRAS (*n*=230), bHLH (*n*=176), AP2/ERF-ERF (*n*=171), MYB (*n*=151), WRKY (*n*=149), C2C2 (*n*=142), MYB-related (*n*=138), NAC (*n*=134), GARP-G2-like (*n*=117), and bZIP (*n*=109) (Fig. 6a).

In addition, the variations in the expression levels of the TFs between these groups were also examined. Figure 6b demonstrates that 26 DEGs from 9 TF families were upregulated under drought stress, of which the GRAS (ten), bHLH (three), and AP2/ERF-ERF (three) families made up a sizable fraction. Strikingly, the FPKM values of most GRAS, AP2/ERF-ERF, MYB, and WRKY TFs were larger in the B group than in the other two groups, especially GRAS TFs (such as *SMil-00017834-mRNA-1* and *SMil-00013932-mRNA-1*).

### Identification of differentially expressed metabolites under different degrees of drought treatments

**Fig. 7:**
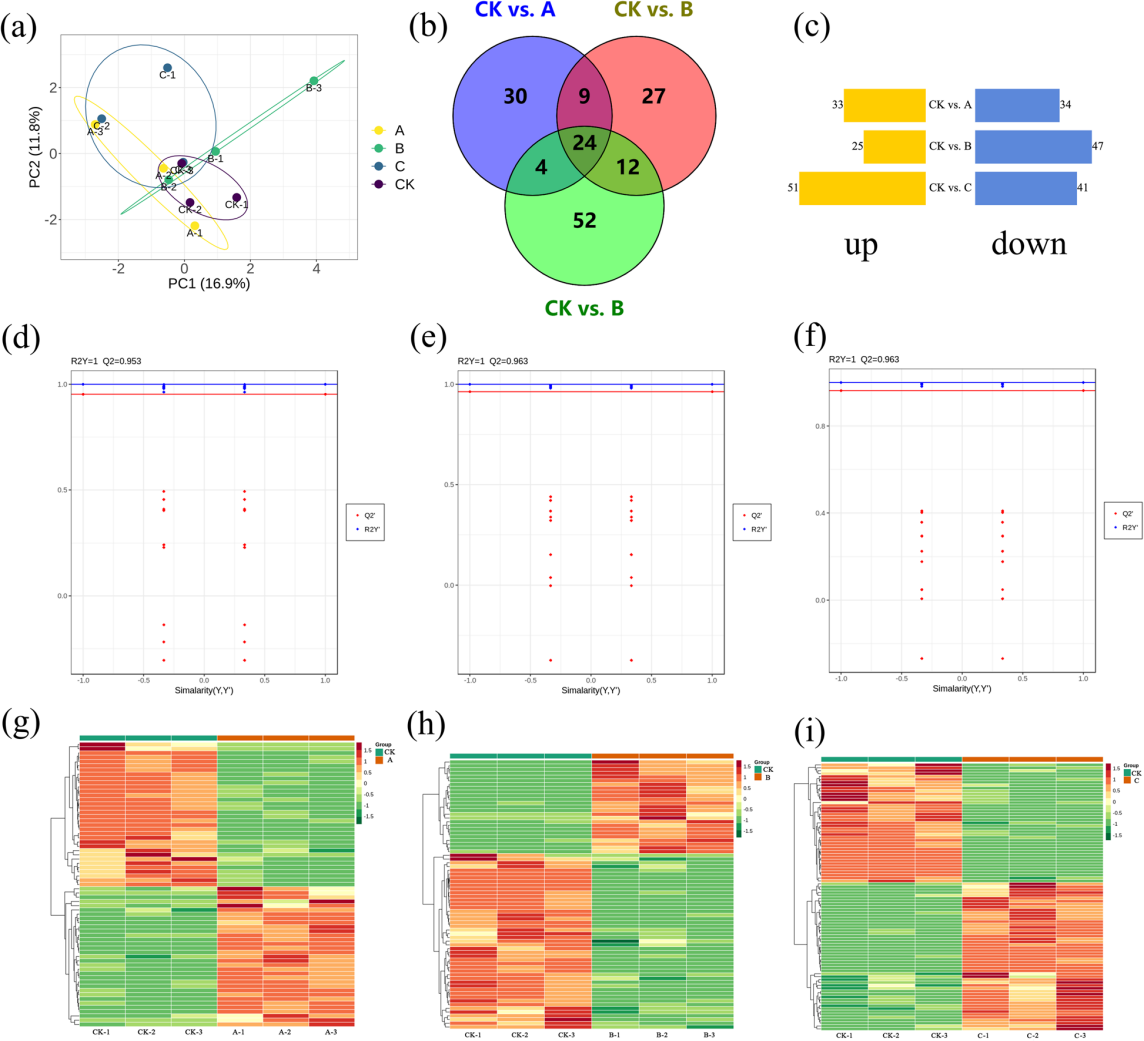
Widely targeted metabolome analysis of *S. miltiorrhiza* after distinct degrees of drought treatments. **a** PCA. **b**, **c** Venn diagram of the up-regulated and down-regulated DAMs. **d**, **e**, **f** Permutation of OPLS-DA for the A, B, and C groups, respectively. R2Y indicates the interpretation rate of Y matrices. Q2 represents the predictive power of the model. **g**, **h**, **i** DAM clustering heatmaps for the A, B, and C groups in comparison to the control. Red and green are employed to symbolize high and low abundance, respectively

With the aid of the UPLC-MS/MS platform, a broadly targeted metabolome analysis was carried out to identify the metabolite changes that *S. miltiorrhiza* underwent after different degrees of drought treatments. First, the variations in metabolites were explored using PCA and orthogonal projection to latent structures-discriminate analysis (OPLS-DA). The results indicated that there was differentiation between the control and drought-treated groups, implying the stability and replicability of the detection method. In addition, OPLS-DA score permutations were obtained, and evident differences were observed for CK vs. A (R2Y=1, Q2=0.953), CK vs. B (R2Y=1, Q2=0.963), and CK vs. C (R2Y=1, Q2=0.963), demonstrating the suitability of the constructed model. These results indicated that the metabolite profile changed under different drought stresses (Fig. 7a, d, e, f). Then, the DAMs were further screened using the changes in metabolites under the different degrees of drought stress (Fig. 7g, h, i). The disparities in DAMs among these groups were also illustrated using Venn diagrams. These groups identified 24 substances, including flavones (chrysin O-malonylhexoside and O-methylchrysoeriol 5-O-hexoside) and terpenes (phytocassane D). Compared with the control group, 67 DAMs were found in group A, 72 in group B and 92 in group C (Fig. 7b, c, Table S 5).

### KEGG enrichment analysis of DAMs

**Fig. 8:**
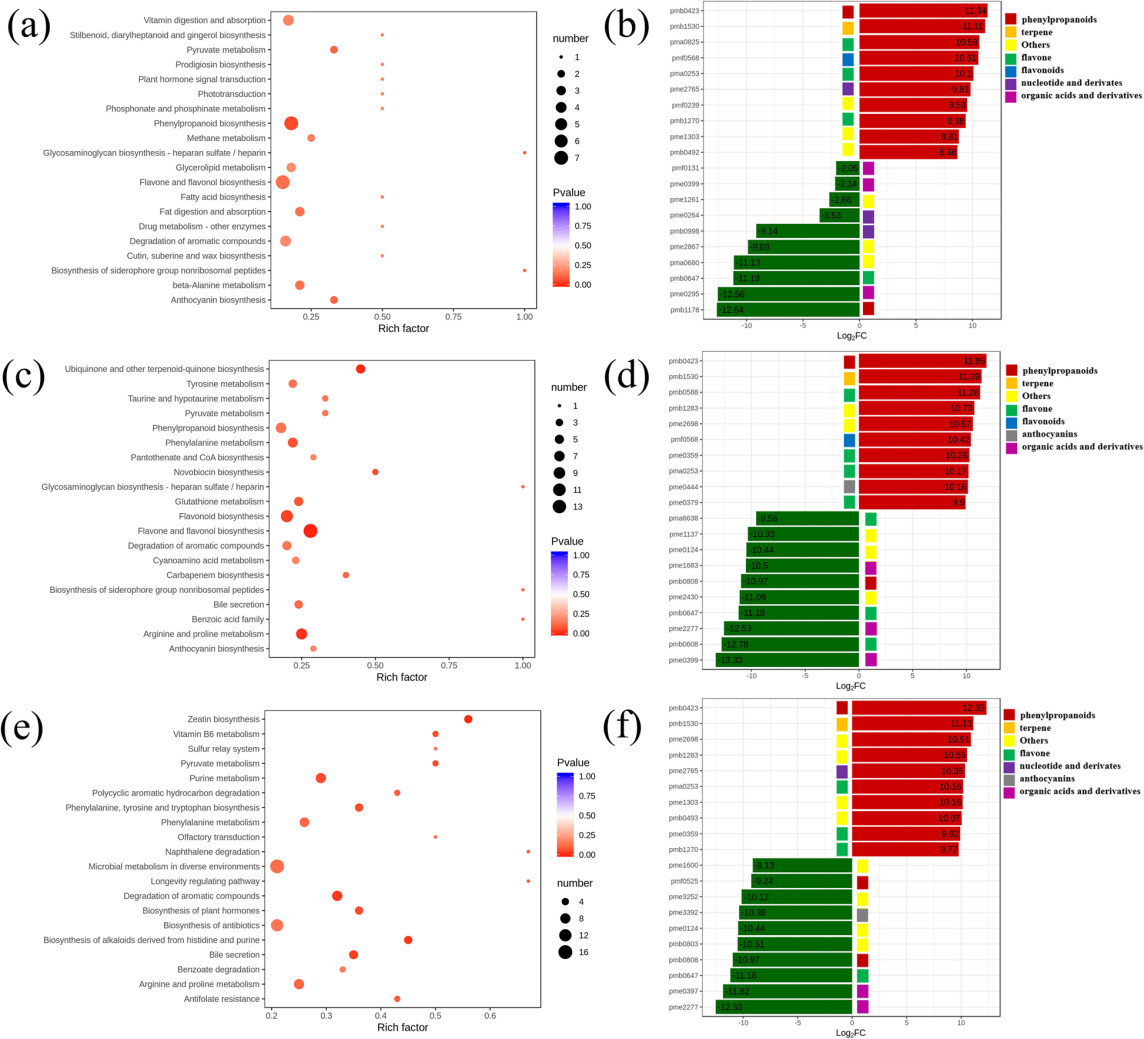
Statistical analysis of DAMs under different degrees of drought treatment. (a, c, d) KEGG analysis of DAMs in CK vs. A, CK vs. B, and CK vs. C, respectively. The significance of the enrichment increases as the *p* value approaches zero. **b, d, f** The |log_2_FC| of the top 20 significantly changed metabolites in these groups. The images are used and adapted with the permission of Kanehisa Laboratories

In this research, the rich factor, *p* value, and quantity of enriched metabolites were used in the KEGG analysis to expand our understanding of the functions of DAMs. The results revealed that most of them were enriched in flavone and flavonol and phenylpropanoid biosynthesis among CK vs. A (Fig. 8a), phenylpropanoid biosynthesis, flavonoid biosynthesis, tyrosine, arginine and proline metabolism, glutathione metabolism, and ubiquinone and other terpenoid-quinone biosynthesis in CK vs. B (Fig. 8c), and phenylalanine metabolism, biosynthesis of antibiotics, purine, arginine and proline metabolism in CK vs. C (Fig. 8 e). When comparing different degrees of drought stress treatment with the control, the top 20 DAMs were displayed using the order of |log_2_FC| (Fig. 8b, d, and f). The most significant DAM was pmb0423 (hydroxy-methoxycinnamate), with a log_2_FC of 11.34 among the CK group vs. A, 11.86 in the CK group vs. B, and 12.33 in the CK group vs. C. After comparison with the database, hydroxy-methoxycinnamate belonged to the phenylpropanoids. In addition, the overproduced DAMs also included terpenes and flavonoids. These results suggested that there was an extensive accumulation of secondary metabolites and that these substances may be essential in *S. miltiorrhiza*’s response to drought stress.

### Response of *S. miltiorrhiza* induced by drought stress

**Fig. 9:**
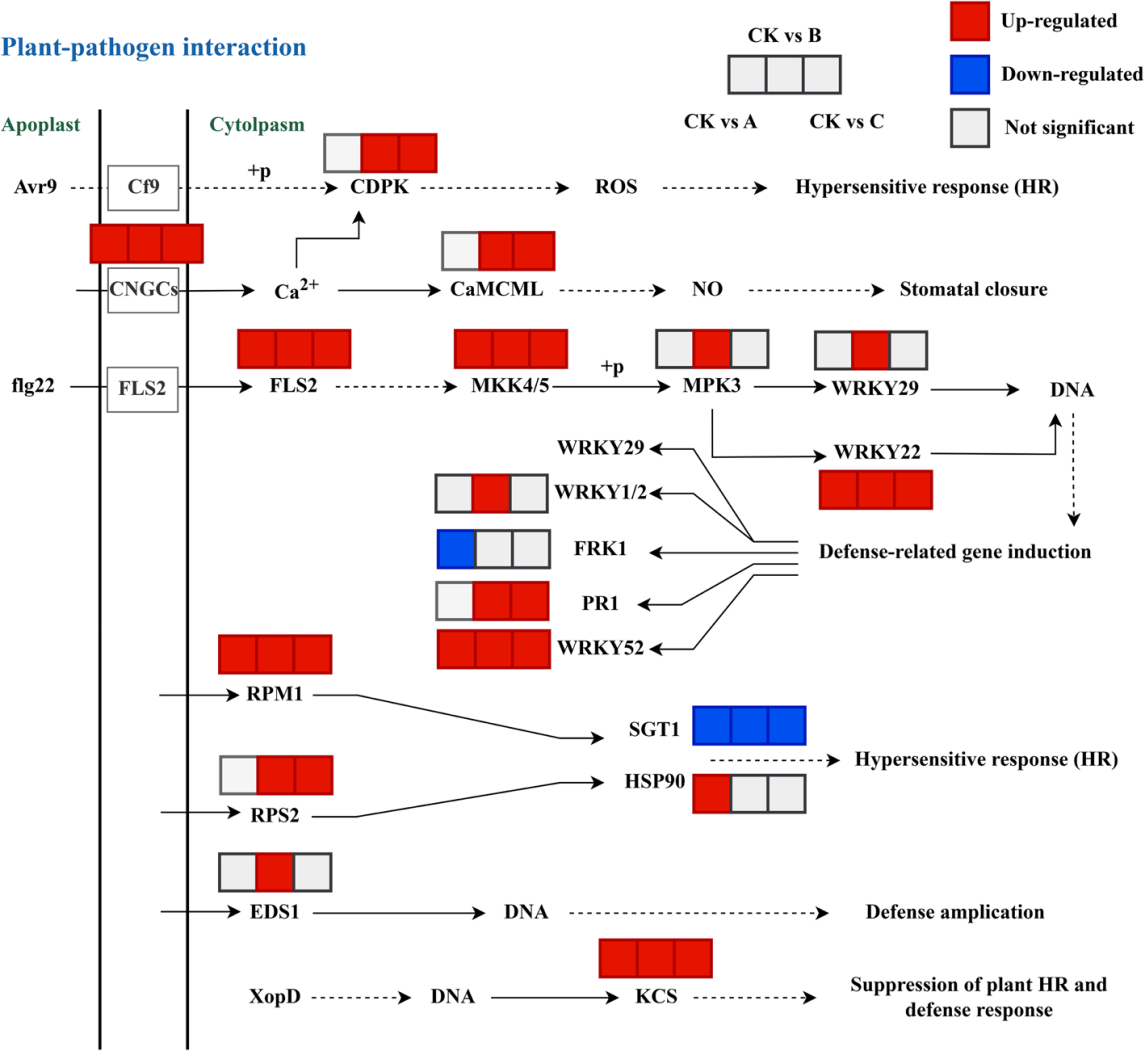
DEGs in plant-pathogen interactions under different degrees of drought stress. Avr9: race-specific elicitor A9; Cf9: disease resistance protein; CDPK: calcium-dependent protein kinase; CNGCs: cyclic nucleotide gated channel; CaMCML: calmodulin; flg22: flagellin; FLS2: flagellin sensitive 2; MKK4/5: mitogen-activated protein kinase kinase 4/5; MPK3: mitogen-activated protein kinase 3; WRKY29/22/1/2/52: WRKY transcription factor 29/22/1/2/52; FRK1: senescence-induced receptor-like serine/threonine-protein kinase; PR1: pathogenesis-related protein 1; RPM1/RPS2: disease resistance protein; SGT1: suppressor of G2 allele of SKP1; HSP90: heat shock protein 90 kDa beta; EDS1: enhanced disease susceptibility 1 protein; XopD: type III effector protein; KCS: 3-ketoacyl-CoA synthase

**Fig. 10:**
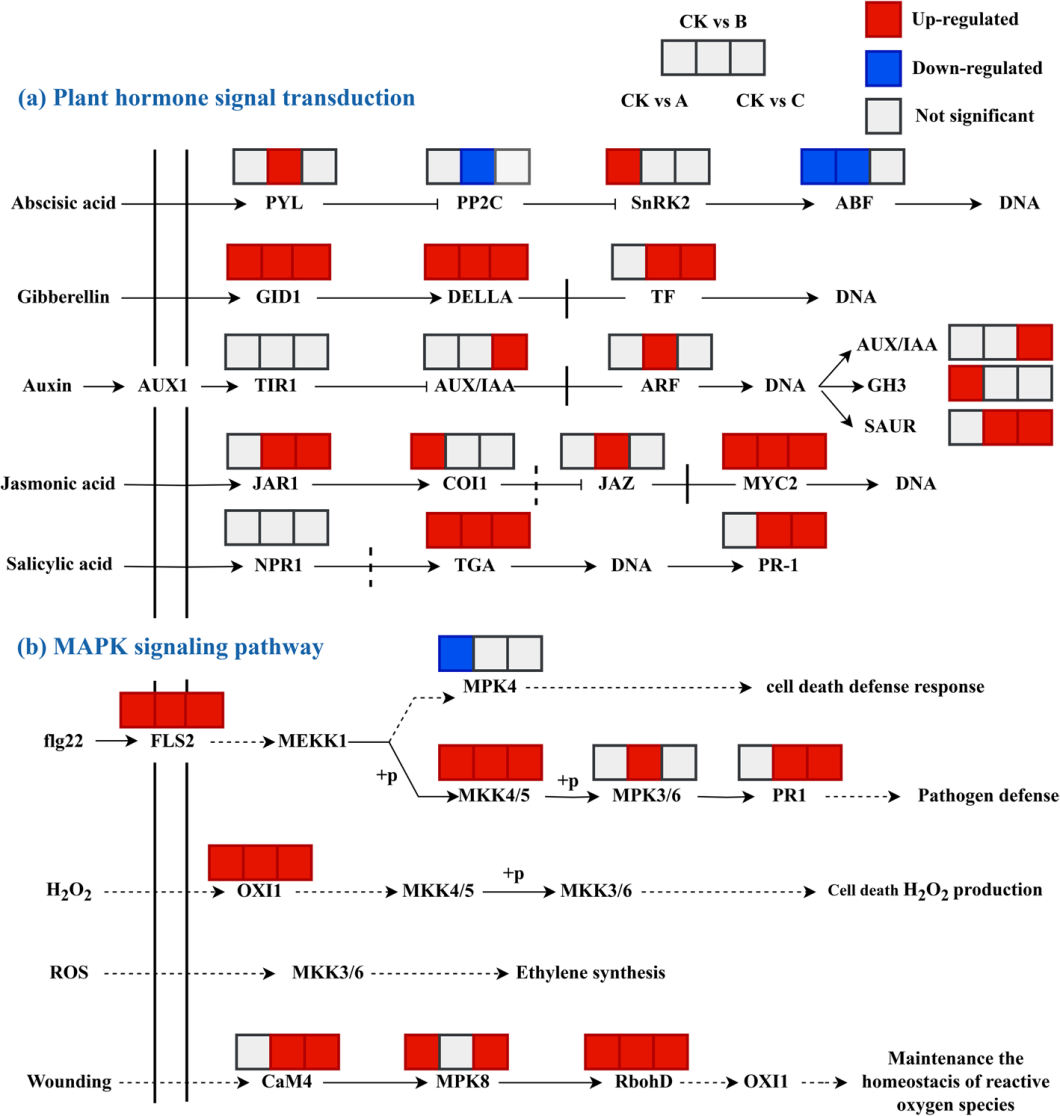
DEGs in plant hormone signal transduction and the MAPK signaling pathway under different degrees of drought stress. PYL: abscisic acid receptor PYL family; PP2C: protein phosphatase 2C; SnRK2: serine/threonine-protein kinase; ABF: ABA responsive element binding factor; GID1: gibberellin receptor; DELLA: DELLA protein; TF: phytochrome-interacting factor 4; AUX/IAA: auxin-responsive protein; ARF: auxin-responsive protein; GH3: auxin responsive gene; SAUR: SAUR family protein; JAR1: SAUR family protein; JAZ: jasmonate ZIM domain-containing protein; MYC2: transcription factor; COI1: coronatine-insensitive protein 1; NPR1: regulatory protein; TGA: transcription factor; PR-1: pathogenesis-related protein 1; MEKK1: mitogen-activated protein kinase kinase kinase 1; MPK4: mitogen-activated protein kinase 4; MKK4/5: mitogen-activated protein kinase kinase 4/5; MPK3/6: mitogen-activated protein kinase 3; OXI1: serine/threonine-protein kinase OXI1; CaM4: calmodulin; MPK8: mitogen-activated protein kinase 8; RbohD: respiratory burst oxidase

**Fig. 11:**
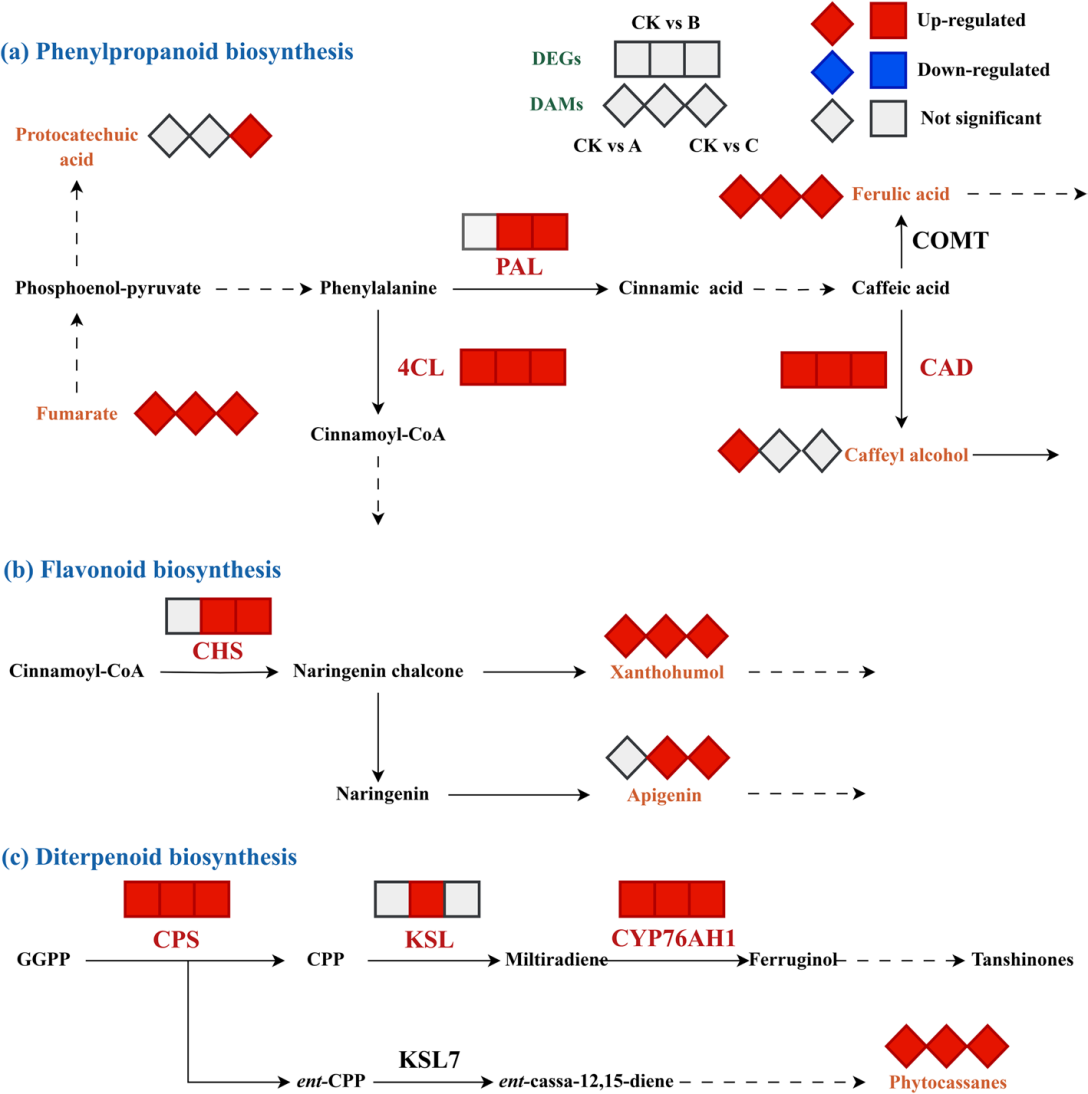
DEGs involved in phenylpropanoid biosynthesis, flavonoid biosynthesis, and diterpenoid biosynthesis under different degrees of drought stress. DEGs were selected based on |log_2_FC| ≥ 1. PAL: phenylalanine ammonia-lyase; 4CL: 4-coumarate--CoA ligase; CAD: cinnamyl-alcohol dehydrogenase; CHS: chalcone synthase; CPS: ent-copalyl diphosphate; KSL: ent-kaurene synthase; CYP76AH1: ferruginol synthase

While comprehensively analysing these enrichment results, we also discovered, quite interestingly, that numerous DEGs and DAMs were related to plant-pathogen interactions, the MAPK signaling pathway, phenylpropanoid, flavonoid, and diterpenoid biosynthesis and plant hormone signal transduction compared with those in the control group. Next, we discussed these pathways thoroughly (Figs. 9, 10, 11).

One of the major metabolic pathways with significantly greater DEG enrichment in these groups was the “plant-pathogen interaction and MAPK signalling” pathway. Additionally, the “plant hormone signal transduction” pathway was remarkably differentially abundant between CK and B groups. To explore the connection between these pathways and the response of *S. miltiorrhiza* to drought stress, the genes related to these pathways were investigated. For the plant-pathogen interaction pathway, 18 enzymes were annotated, including 16 up-regulated and 2 down-regulated enzymes. Furthermore, the DEGs encoding CNGCs, FLS2, MKK4/5, RPM1, KCS, and WRKY22/52 were significantly upregulated in these three comparison groups. The DEGs encoding CDPK, CaMCML, PR1, and PRS2 were only upregulated in CK vs. B, suggesting that these DEGs changed significantly when the soil water content was 65% (B group) (Fig. 9).

Among the enrichment results, 9 enzymes related to the “MAPK signaling pathway” and 20 enzymes connected to “plant hormone signal transduction” were uncovered. As shown in Fig. 10a, in detail, the DEGs encoding AUX/IAA, ARF, and SAUR in the auxin signaling pathway were significantly upregulated. The DEGs connected to ABA signal transduction, such as soluble ABA receptors pyrabactin resistance 1 (PYR1)-like (PYL) and sucrose non-fermenting 1-related protein kinase 2 (SnRK2), were upregulated under drought stress; however, the opposite was observed for protein phosphatases type-2C (PP2Cs). In addition, DEGs encoding TGA and PR-1, which are involved in SA signaling, and GID1, DELLA and TF, which are involved in GA signaling, were upregulated under drought stress. Furthermore, during the MAPK signaling pathway, the DEGs encoding FLS2, MKK4/5, OXI1, and RbohD were significantly upregulated, whereas the opposite was found for genes involved in MPK4 (Fig. 10b).

The important secondary metabolism in plants includes the biosynthesis of phenylpropanoids and flavonoids. Furthermore, diterpenoid biosynthesis is one of the important pathways in *S. miltiorrhiza* and is closely related to the active ingredient*.* Among the annotated results of DEGs and DAMs in the phenylpropanoid biosynthesis pathway, DEGs encoding PAL, 4CL, and CAD were significantly upregulated. The changes in compounds, including fumarate, ferulic acid, and caffeyl alcohol, were also significantly upregulated (Fig. 11a). As shown in Fig. 11b, there was 1 enzyme and 2 compounds involved in flavonoid biosynthesis, and the DEGs encoding CHS were upregulated in the B and C groups in comparison to the CK group. In addition, we found that the accumulation of xanthohumol and apigenin was significantly increased after drought stress and that the accumulation of apigenin was in line with the CHS gene. The DEGs encoding CPS and CYP76AH1 were considerably upregulated among diterpenoid biosynthesis genes, indicating that drought stress may be related to the biosynthesis of tanshinones. Moreover, we found that one metabolite, phytocassanes, was significantly upregulated in all of the groups (Fig. 11c).

Synchronously, for the sake of verifying the dependability of the RNA-seq data, we randomly chose 10 DEGs to confirm the sequencing results, including the key rate-limiting enzyme GGPPS (*SMil-00019981-mRNA-1*) downstream of the pathway. Similar with the results of the RNA-seq approach, the 10 DEGs discovered by qRT-PCR differed significantly under the different degrees of drought stress (Figure S 4).

### Correlation analysis

**Fig. 12:**
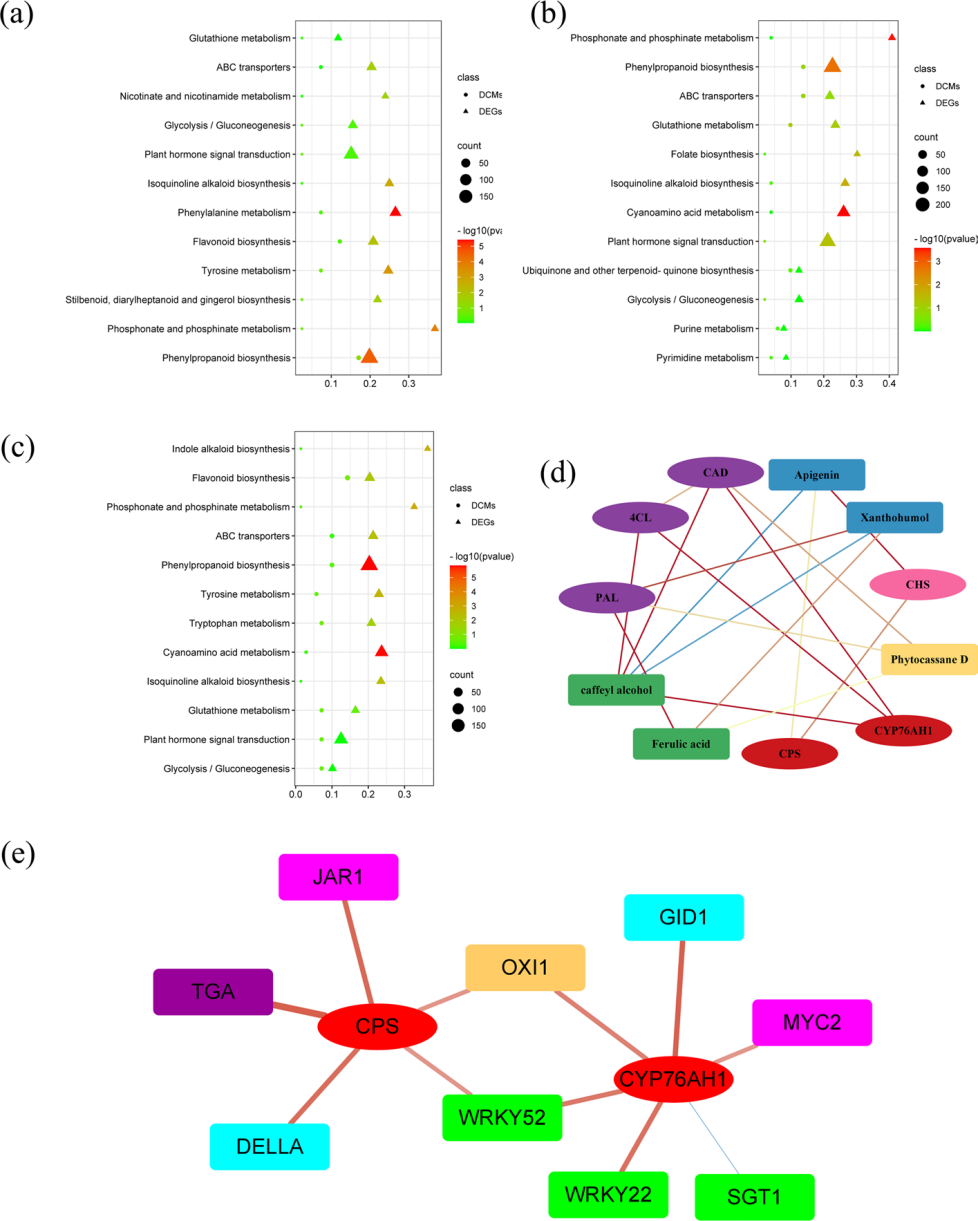
Correlation analysis between DEGs and DAMs. **a**-**c** Graphs of DEGs and DAMs enriched along the same KEGG pathway among these three groups in comparison to the control. **d** Coexpression network analysis between DEGs and DAMs. The red and yellow shapes represent the enzymes and compounds involved in the diterpenoid biosynthesis pathway, respectively. The pink and blue shapes represent the enzymes and compounds involved in the biosynthesis pathway of flavonoids, respectively. The purple and green shapes represent the enzymes and compounds implicated in the phenylpropanoid biosynthesis pathway, respectively. Red lines indicate a positive correlation with R > 0.9. The yellow lines indicate a positive correlation with R > 0.8. The blue lines indicate a negative correlation with R > 0.9. PAL: phenylalanine ammonia-lyase; 4CL: 4-coumarate--CoA ligase; CAD: cinnamyl-alcohol dehydrogenase; CHS: chalcone synthase; CPS: ent-copalyl diphosphate; CYP76AH1: ferruginol synthase. **e** Coexpression network analysis between DEGs. The red circles represent the enzymes in the diterpenoid biosynthesis pathway. The pink (related to JA hormone), blue (related to GA hormone), and purple (related to SA hormone) shapes represent the enzymes involved in the plant hormone signal transduction. The green shapes represent the enzymes implicated in plant-pathogen interactions. The orange shapes represent the enzymes implicated in the MAPK signaling pathway. The thicker the line and the closer the color is to red, the stronger the correlation is ( R > 0.8). The blue lines indicate a negative correlation with R > 0.6

Combined transcriptome and metabolomic analysis was conducted to learn more about the function of DEGs and DAMs of *S. miltiorrhiza* after different degrees of drought stress. Many DEGs and DAMs were abundant in the same KEGG pathways, including phenylpropanoid biosynthesis, cyanoamino acid metabolism, flavonoid biosynthesis, ABC transporters, and plant hormone signal transduction (Fig. 12a, b, c). According to these findings, these metabolite changes may be regulated by the respective genes either directly or indirectly, and they may be closely related to how *S. miltiorrhiza* reacts to drought stress.

Based on the Pearson correlation coefficient, a correlation network graph of DEGs and DAMs was constructed to further investigate the gene regulatory network of *S. miltiorrhiza* under drought stress. The results of the correlation study revealed that PAL, 4CL, and CAD were highly positively connected to phenylpropanoid contents and that CHS was highly positively connected to apigenin, one of the flavonoids. Intriguingly, CYP76AH1 implicated in the diterpenoid biosynthesis pathway was positively connected to PAL, 4CL, and CAD (Fig. 12d). These findings suggested that these genes might be essential for *S. miltiorrhiza* to enhance drought resistance and promote the accumulation of tanshinones.

To further explore whether genes in other pathways (plant-pathogen interactions, the MAPK signaling pathway, and plant hormone signal transduction) also had dual roles in response to drought stress and tanshinone regulation, we constructed a correlation network graph of DEGs involved in these pathways (Fig. 12e). We found that WRKY22/WRKY52 involved in the plant-pathogen interactions and GID1 involved in the plant hormone signal transduction were highly positively connected to CYP76AH1 gene. JAR1, DELLA, and TGA involved in plant hormone signal transduction were highly positively connected to CPS gene. All of the correlations were greater than 0.8. These results indicated that these genes might function in response to drought stress and promote the accumulation of tanshinones in *S. miltiorrhiza.*

## Discussion

**Fig. 13:**
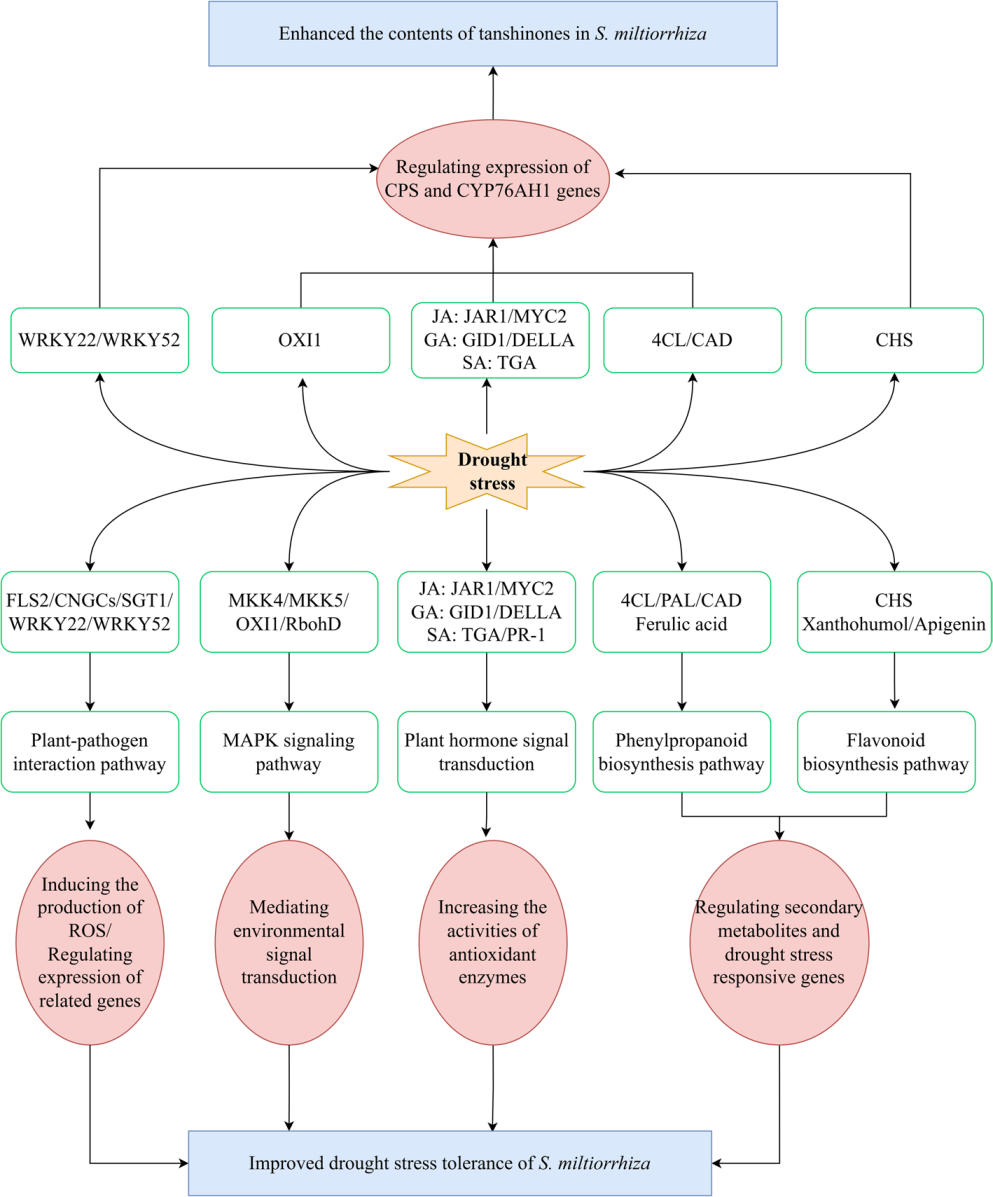
Illustrated depiction summarizing the main pathways under drought stress of *S. miltiorrhiza*

The dry root and rhizome of *S. miltiorrhiza* is popular among traditional Chinese medicine because of its strong pharmacological and therapeutic effects [40–42]. In recent years, the yield of *S. miltiorrhiza* has been negatively impacted by increasingly harsh environmental conditions, particularly drought stress. Numerous studies have demonstrated that the response mechanisms of different species under drought stress are also different. In our study, transcriptome data were combined with widely targeted metabolite profiles to investigate the drought response mechanism of *S. miltiorrhiza*. Furthermore, we found that there were significant differences in plant-pathogen interactions, the MAPK signaling pathway, the biosynthesis of phenylpropanoids, flavonoids, and diterpenoids and plant hormone signal transduction (Fig. 13).

### Plant-pathogen interaction in response to drought stress

Plant-pathogen interactions, which exhibit two patterns, PAMP-triggered immunity (PTI) and effector-triggered immunity (ETI), are crucial physiological processes that occur in plants [43]. It has been reported that drought stress could trigger the plant-pathogen interaction and regulate the expression of genes implicated in this pathway, such as FLS2, CNGCs and SGT1 [44]. FLS2 (flagellin sensitive 2), which exists in the plasma membrane, is one of the immune signal receptors participating in the plant-pathogen interaction pathway and can cause a decline in ROS [45, 46]. Studies have demonstrated that FLS2 works together with RBOHD and PIF4 to respond to drought stress [47]. Here, we discovered that the DEGs encoding FLS2 were significantly upregulated in comparison to the control group (Fig. 9), indicating that FLS2 may be a key player in the response to drought stress. Eukaryotes have cyclic nucleotide-gated channels (CNGCs), which act as calcium sensors. Calcium is crucial not only for plant growth and development but also for drought, salt stress, and disease resistance [48]. In our study, the DEGs encoding the CNGCs dramatically increased under drought stress, which was in line with the results of *CsCNGC1.4/2.1/4.2* in *Citrus sinensis* [49]. Interestingly, we also found that the DEGs encoding one suppressor of the G2 allele of skp1 (SGT1) were remarkably downregulated between these treatment groups. Furthermore, research has shown that OsSGT1 is important for the response of rice to drought [50]. Based on this, we made the assumption that SGT1 might react negatively to drought stress. In addition, drought stress could modulate WRKY TFs, such as WRKY22 [51]. WRKY22 and WRKY52, significantly increased after drought treatment in our research, implying that these two genes may be connected to drought resistance.

### Response of the MAPK signaling pathway to drought stress

One of the most well-researched plant signaling pathways, the MAPK signaling pathway, is composed of a class of protein kinases that are crucial for stress responses [52]. Studies have reported that the MAPK signaling pathway could be activated by drought stress, and MAPK pathway genes improved drought tolerance [53, 54]. In our investigation, DEGs encoding MKK4, MKK5, OXI1, and RbohD were significantly upregulated in the A, B, and C groups compared to the control group. MKK4/5 protein kinases are mitogen-activated protein kinase kinases that mediate environmental signal transduction to induce stress responses. Under drought stress, excessive ROS production induces oxidative stress, which eventually results in cell membrane rupture and stimulates various stress signaling pathways, including the MAPK signaling pathway [55, 56]. OXI1 kinase is necessary for MAPK protein kinase activation and is an integral component of the signal transduction pathway that transmits the signal to a variety of downstream reactions [57]. In addition, RbohD (respiratory burst oxidase) is another key gene involved in the MAPK signaling pathway, and it could cooperate with other genes to respond to drought stress [47]. Therefore, we conjectured that MKK4/5 might be significant in improving the drought resistance ability of *S. miltiorrhiza*.

### Response of plant hormones to drought stress

It is well recognized that plant hormones play significant roles in modulating the plant defence response to drought stress. Key hormones produced by plants include ABA, JA, GA, SA, and auxin [13]. Numerous studies have indicated that the accumulation of JA could trigger downstream TFs and subsequently respond to stress [58]. Among this pathway, a previous study suggested that JAR1-mediated could improve drought stress tolerance of *Arabidopsis* [59]. According to our findings, the DEGs encoding JAR1 significantly increased in groups B and C. In addition, it has been noted that during drought stress, the transcription level of *SlMYC2* considerably increased [60]. These results, which were in line with a previous study, indicated that the expression of the DEG encoding MYC2 was markedly upregulated in both groups. This finding revealed that the JA hormone might be crucial for the drought stress response of *S. miltiorrhiza*. Generally, the main functions of SA are regulating physiological processes, including photosynthesis and the activity of antioxidant enzymes [61]. Under drought stress, the DEGs encoding TGA and PR-1 were shown to be upregulated in this study; synergistically, it was discovered that the activity of antioxidant enzymes was boosted, especially POD activity (Fig. 1c). Thus, the SA hormone might increase the ability of *S. miltiorrhiza* to withstand drought by enhancing the activity of antioxidant enzymes.

GA hormone is also crucial in mediating the stress response. Under drought stress, GID1, one of the GA receptors, was notably upregulated in *Elymus sibiricu*s [62]. In tomatoes, DELLA proteins help tolerate drought stress [63]. It was also interesting to note that, in line with earlier findings, the DEGs encoding GID1 and DELLA were notably upregulated. These results imply that GA might improve the capacity of *S. miltiorrhiza* for drought stress adaptation. Moreover, drought stress could also stimulate ABA accumulation. For example, in *P. nutans*, PYL, PP2C, and SnRK2 were notably upregulated [64]. DEGs that encode PYL, PP2C, and SnRK2 in our research, however, showed a decrease or no significant change. We speculated that this result may be due to species differences and different methods of drought stress treatment.

### Analysis of transcription factors

TFs are crucial for the response to drought stress and the regulation of secondary metabolites [64, 65]. Previous results have proven that some TFs function through the MAPK signaling pathway and plant hormone signal transduction. To elucidate the biological functions of TFs in *S. miltiorrhiza* under drought stress, TFs expressed in both drought stress groups were determined. Forty-five DEGs from 14 TF families, including GRAS, bHLH, AP2/ERF-ERF, MYB, and WRKY, were annotated in this work (Fig. 6). *CaGRAS* 12 has been shown to be a drought-responsive gene, making it a possible candidate gene for improving drought tolerance in *Cicer arietinum* [66]. Previous results revealed that *SlbHLH96* could mediate drought resistance in tomatoes [67]. Additionally, more TF genes showed differential expression under drought stress in *Medicago sativa* [68]. With these differential results, we hypothesized that these annotated TFs might play a significant regulatory function in *S. miltiorrhiza* to enhance drought tolerance.

### Secondary metabolism induced by drought stress

Plant secondary metabolites are unique resources that are widely used to make medicines, food additives, and biochemicals with significant commercial applications. In general, they are essential for plants to adapt to their environment and deal with stress [69]. Nevertheless, many studies have demonstrated that environmental elements such as drought stress may have a significant impact on secondary metabolites. In *Casuarina equisetifolia*, drought stress elevated the expression of associated genes and increased the accumulation of flavonoids and phenols [19]. In *Salvia officinalis*, the amount of monoterpenes significantly increased in response to drought stress [68]. The concentrations of most phenolic and flavonoid components increased with the aggravation of drought severity in *Achillea pachycephala* Rech.f. [70]. Research has also shown that a large variety of secondary metabolites are produced by general phenylpropanoid metabolism and that the phenylpropanoid pathway is activated under drought stress [71, 72]. In our study, there was an extensive accumulation of compounds, including fumarate, ferulic acid, xanthohumol, and apigenin, under drought stress (Fig. 11a, b). Furthermore, the DEGs encoding PAL, 4CL, CAD, and CHS were significantly upregulated. Intriguingly, we found that the trend between CHS and apigenin was consistent, and correlation analysis showed that CHS was strongly positively connected to apigenin. Studies have shown that CHS is an essential rate-limiting enzyme in the pathway that produces flavonoids and is necessary for regulating plant growth, development, and abiotic stress resistance [73]. All of these results supported earlier research and indicated that the biosynthesis pathways of phenylpropanoids and flavonoids, especially CHS genes and apigenin, were involved in the response of *S. miltiorrhiza* to drought stress [20].

In addition, we found one increased metabolic compound, phytocassanes, which is involved in the diterpenoid biosynthesis pathway and may be connected to the response to drought stress. Phytocassanes are usually protective substances produced when plant tissues are destroyed and damaged by foreign pathogens and microorganisms. Therefore, we hypothesized that the tissues of *S. miltiorrhiza* plants were destroyed and that this kind of substance was produced to enhance drought resistance. As one of the most significant secondary metabolites, tanshinones are frequently utilized to treat cardiovascular and cerebrovascular diseases [27, 28]. Interestingly, CYP76AH1, which is associated with the biosynthesis of tanshinones, was strongly connected to PAL, 4CL, and CAD, which are involved in the phenylpropanoid biosynthesis pathway (Fig. 12d). Based on these results, we inferred that the phenylpropanoid metabolic pathway was not only closely related to the drought stress response but also may be related to the biosynthesis of tanshinones.

Interestingly, we also found that DEGs (WRKY22, WRKY52, JAR1, DELLA, TGA, and GID1) involved in plant-pathogen interaction, plant hormones were not only upregulated under drought stress, but also strongly connected to the key enzyme genes related to tanshinones biosynthesis (Figs 10 and 12e). Studies have shown that the SA treatment could promote the tanshinone accumulation through the *SmGGPPS*, *SmCPS*, and *SmKSL* genes [74]. JA could regulate the biosynthesis of tanshinones via the JAZ9-MYB76 complex in *S. miltiorrhiza* [75]. And GA could promote hairy roots growth and increase the contents of tanshinones [76]. Therefore, we speculated that these genes have dual functions of responding to drought stress and regulating the accumulation of tanshinones. All of these hypothesis needs to be further verified.

## Conclusion

In our research, transcriptome and metabolomic analyses were combined to investigate the molecular pathways behind *S. miltiorrhiza’*s response to moderate drought stress. Moderate drought stress led to the accumulation of phenylpropanoids, flavonoids, and diterpenoids, including fumarate, ferulic acid, xanthohumol, apigenin, and phytocassanes, which could protect the *S. miltiorrhiza* plant from adverse factors, thereby improving its resistance to drought. The DEGs, especially WRKY22, WRKY52, GID1, JAR1, DELLA, and TGA, involved in plant-pathogen interaction, phenylpropanoid and flavonoid biosynthesis, and plant hormone signal transduction may have the dual functions of responding to drought stress and regulating the accumulation of tanshinones. The specific regulatory mechanism will be the focus of future research. This research provides a theoretical foundation for studying the genetic regulation of drought stress tolerance and tanshinones accumulation. Simultaneously, this will facilitate further study of the more complex regulatory mechanisms of *S. miltiorrhiza* under moderate drought stress.

### Supplementary Information

**Additional file 1:** **Table S1.** The measurement method of physiological indexes. **Table S2.** The primer sequences used in the real-time PCR. **Table S3.** Evaluation statistics of sequencing data of root samples of *Salvia miltiorrhiza. ***Table S4.** Top 15 up-down DEGs statistics. **Table S5.** Up-down DAMs statistics. **Table S6.** The FPKM of 28 TFs. **Fig S1.** The heat map analysis showed that the correlation coefficients of all groups. **Fig S2.** The PCA analysis showed that the differences are significant in the expression of Unigenes under different drought stress treatments. **Fig S3.** Top 20 GO enrichment pathways involved in different degrees of drought stress of *S. miltiorrhiza*. **Fig S4.** Real-time PCR validation of DEGs in the group CK, A, B, and C respectively. Values are as means ±SD, *n*=3. “**” represents *p *< 0.01, “*” represents *p *< 0.05.

## Data Availability

The sequenced raw reads generated in this study have been submitted to the National Center for Biotechnology Information (NCBI) with BioProject ID: PRJNA1025047 (https://dataview.ncbi.nlm.nih.gov/object/PRJNA1025047)
