# Supplementary material for: Transcriptome sequencing and metabolome analysis reveal the molecular mechanism of Salvia miltiorrhiza in response to drought stress
Source: BMC Plant Biol. 2024 May 23;24:446. doi: 10.1186/s12870-024-05006-7 (PMC11112794; doi:10.1186/s12870-024-05006-7)
Supplement: Supplementary file 1 — Additional file 1: Table S1. The measurement method of physiological indexes. Table S2. The primer sequences used in the real-time PCR. Table S3. Evaluation statistics of sequencing data of root samples of Salvia miltiorrhiza. Table S4. Top 15 up-down DEGs statistics. Table S5. Up-down DAMs statistics. Table S6. The FPKM of 28 TFs. Fig S1. The heat map analysis showed that the correlation coefficients of all groups. Fig S2. The PCA analysis showed that the differences are significant in the expression of Unigenes under different drought stress treatments. Fig S3. Top 20 GO enrichment pathways involved in different degrees of drought stress of S. miltiorrhiza. Fig S4. Real-time PCR validation of DEGs in the group CK, A, B, and C respectively. Values are as means ±SD, n=3. “**” represents p < 0.01, “*” represents p < 0.05. [file 12870_2024_5006_MOESM1_ESM.zip › Supplementary file/Supplementary Figures.doc]

**Supplementary figures in the paper**

**Figure S1. The heat map analysis of correlation coefficients in all groups.** The closer the color is to pink, the stronger the correlation is. The closer the color is to green, the weaker the correlation is.

**Figure S2. The principal component analysis (PCA) of Unigenes under different drought stress treatments.**

**Figure S3. Top 20 GO enrichment pathways involved in different degrees of drought stress of *S. miltiorrhiza*.** (a, b, c) The DEGs involved in the biological process (BP), cellular component (CC) and molecular function (MF) in the CK vs. A, respectively. (d, e, f) The DEGs involved in the biological process (BP), cellular component (CC) and molecular function (MF) in the CK vs. B, respectively. (g, h, i) The DEGs involved in the biological process (BP), cellular component (CC) and molecular function (MF) in the CK vs. C, respectively.

**Figure S4. Real-time PCR validation of DEGs in the group CK, A, B, and C respectively.** Values are as means ±SD, n=3. “**” represents *p* < 0.01, “*” represents *p* < 0.05.
