# Supplementary material for: Transcriptome sequencing and metabolome analysis reveal the molecular mechanism of Salvia miltiorrhiza in response to drought stress
Source: BMC Plant Biol. 2024 May 23;24:446. doi: 10.1186/s12870-024-05006-7 (PMC11112794; doi:10.1186/s12870-024-05006-7)
Supplement: Supplementary file 1 — Additional file 1: Table S1. The measurement method of physiological indexes. Table S2. The primer sequences used in the real-time PCR. Table S3. Evaluation statistics of sequencing data of root samples of Salvia miltiorrhiza. Table S4. Top 15 up-down DEGs statistics. Table S5. Up-down DAMs statistics. Table S6. The FPKM of 28 TFs. Fig S1. The heat map analysis showed that the correlation coefficients of all groups. Fig S2. The PCA analysis showed that the differences are significant in the expression of Unigenes under different drought stress treatments. Fig S3. Top 20 GO enrichment pathways involved in different degrees of drought stress of S. miltiorrhiza. Fig S4. Real-time PCR validation of DEGs in the group CK, A, B, and C respectively. Values are as means ±SD, n=3. “**” represents p < 0.01, “*” represents p < 0.05. [file 12870_2024_5006_MOESM1_ESM.zip › Supplementary file/Supplementary Table S1-The measurement method of physiological indexes.doc]

1. **The measurement of total protein**

The total protein quantification kit (Coomassie Brilliant Blue) purchased from Jiancheng Bioengineering Institute (Nanjing, China) was used to analyse the changes of total protein. After the samples to be tested were mixed with Coomassie brilliant blue solution and protein standard solution, they were allowed to stand for 10 minutes, and the OD value was measured at 595nm. The calculation formula is as follows:

2. **The measurement of superoxide dismutase (SOD)**

The Copper-zinc superoxide dismutase (CuZn-SOD) assay kit purchased from Jiancheng Bioengineering Institute (Nanjing, China) was used to analyse the changes of SOD activity. Tissue samples were weighed and homogenized (1:10, w/v) in 50 mmol/l phos phate buffer (pH 7.4). The calculation formula is as follows:

$\begin{matrix} T\text{-}SOD\text{ activity}=\frac{\text{control OD value-determined OD value}}{\text{control OD value }}\div50\text{\%}\times\left[\frac{\text{total volume of reaction solution}}{\text{sample volume}}\right]\times\text{sample pretreatment dilution}\left(\text{3-folds}\right)\div\text{concentration of homegenate} \\ \end{matrix}$ $\begin{matrix} \text{CuZn}-\text{SOD activity}=\frac{\text{control OD value-determined OD value}}{\text{control OD value}}\div50\%\times\left[\frac{\text{total volume of reaction solution}}{\text{sample volume}}\right]\times\text{sample pretreatment dilution(2-folds)}\div\text{concentration of homogenate} \\ \end{matrix}$**3. The measurement of peroxidase (POD)**

The [peroxidase assay kit](http://www.njjcbio.com/products.asp?id=423) purchased from Jiancheng Bioengineering Institute (Nanjing, China) was used to analyse the changes of POD activity. The POD activity was measured based on the change of absorbance at 420 nm by catalyzing H_2_O_2_. One unit was defined as the amount of enzyme which was catalyzed and generated 1 mg substrate by 1.0 g fresh tissues in the reaction system at 37 ℃. The calculation formula is as follows:

$\text{POD activity}=\frac{\text{determined OD value-control OD value}}{12\times\text{specific light diameter}}\times\frac{\text{total volume of reaction solution}}{\text{sample volume}}\div\text{response time}\div\text{protein concentration in the homogenate}\times1000$**4. The measurement of catalase (CAT)**

The catalase (CAT) assay kit (Visible light) purchased from Jiancheng Bioengineering Institute (Nanjing, China) was used to analyse the changes of CAT activity. The CAT activity was measured based on the hydrolysis reaction of hydrogen peroxide (H_2_O_2_) with CAT, which could be terminated by molybdenum acid (MA) to produce yellow MA H_2_O_2_ complex. CAT activity was calculated by the decrease in absorbance at 405 nm due to the degradation of H_2_O_2_, and one unit is defined as the amount of enzyme that will cause the decompose of 1 mmol hydrogen peroxide (H_2_O_2_) per second at 37 ℃ in 1.0 g fresh tissue. The calculation formula is as follows:

$\text{CAT activity}=\left(\text{control OD value-determined OD value}\right)\times271\times\frac{1}{60\times\text{sample volume}}\div\text{protein concentration of samples}$**5. The** **measurement of** **malondialdehyde (MDA)**

The malondialdehyde (MDA) assay kit (TBA method) purchased from Jiancheng Bioengineering Institute (Nanjing, China) was used to analyse the changes of MDA contents. purchased from Jiancheng Bioengineering Institute (Nanjing, China) was used to analyse the changes of CAT activity. The calculation formula is as follows:

$\text{MDA content}=\frac{\text{determined OD value-control OD value}}{\text{standard OD value-black OD value}}\times\text{concentration of standard}\div\text{protein concentration of samples}$**6. The measurement of proline (PRO)**

The proline assay kit purchased from Jiancheng Bioengineering Institute (Nanjing, China) was used to analyse the changes of PRO contents. The plant tissues were precisely weighed and homogenized by adding the corresponding reagents. After centrifugation, the supernatant was taken for determination. The calculation formula is as follows:

$$\begin{matrix} \text{PRO content}=\frac{\text{determined OD value-black OD value}}{\text{standard OD value-black OD value}}\times\text{concentration of standard}\div\text{concentration of homogenate}\times\text{samples tested before dilution} \\ \text{concentration of homogenate}=\frac{\text{weight of tissue}}{\text{total volume of extract}} \end{matrix}$$
